# Disruption of anthrax toxin receptor 1 in pigs leads to a rare disease phenotype and protection from senecavirus A infection

**DOI:** 10.1038/s41598-022-09123-x

**Published:** 2022-03-23

**Authors:** Paula R. Chen, Raymond R. R. Rowland, Ana M. Stoian, Vlad Petrovan, Maureen Sheahan, Charan Ganta, Giselle Cino-Ozuna, Dae Young Kim, James M. Dunleavey, Kristin M. Whitworth, Melissa S. Samuel, Lee D. Spate, Raissa F. Cecil, Joshua A. Benne, Xingyu Yan, Ying Fang, Brad St. Croix, Kelly Lechtenberg, Kevin D. Wells, Randall S. Prather

**Affiliations:** 1 Division of Animal Science, College of Agriculture Food and Natural Resources, University of Missouri, Columbia, Columbia, MO 65211 USA; 2 Department of Diagnostic Medicine and Pathobiology, College of Veterinary Medicine, Kansas State University, Manhattan, KS 66506 USA; 3 Department of Medical Microbiology and Immunology, School of Medicine, University of California Davis, Davis, CA 95616 USA; 4 College of Veterinary Medicine, Kansas State Veterinary Diagnostic Laboratory, Kansas State University, Manhattan, KS 66506 USA; 5 Veterinary Medical Diagnostic Laboratory, College of Veterinary Medicine, University of Missouri, Columbia, MO 65211 USA; 6 Mouse Cancer Genetics Program, Center for Cancer Research, National Cancer Institute, Frederick, MD 21702 USA; 7 Department of Pathobiology, College of Veterinary Medicine, University of Illinois, Urbana, IL 61802 USA; 8 Midwest Veterinary Services, Inc. and Central States Research Centre, Inc., Oakland, NE 68045 USA

**Keywords:** Biotechnology, Cell biology, Molecular biology, Diseases, Pathogenesis

## Abstract

Senecavirus A (SVA) is a cause of vesicular disease in pigs, and infection rates are rising within the swine industry. Recently, anthrax toxin receptor 1 (ANTXR1) was revealed as the receptor for SVA in human cells. Herein, the role of ANTXR1 as a receptor for SVA in pigs was investigated by CRISPR/Cas9 genome editing. Strikingly, *ANTXR1* knockout (KO) pigs exhibited features consistent with the rare disease, GAPO syndrome, in humans. Fibroblasts from wild type (WT) pigs supported replication of SVA; whereas, fibroblasts from KO pigs were resistant to infection. During an SVA challenge, clinical symptoms, including vesicular lesions, and circulating viremia were present in infected WT pigs but were absent in KO pigs. Additional *ANTXR1*-edited piglets were generated that were homozygous for an in-frame (IF) mutation. While IF pigs presented a GAPO phenotype similar to the KO pigs, fibroblasts showed mild infection, and circulating SVA nucleic acid was decreased in IF compared to WT pigs. Thus, this new *ANTXR1* mutation resulted in decreased permissiveness of SVA in pigs. Overall, genetic disruption of *ANTXR1* in pigs provides a unique model for GAPO syndrome and prevents circulating SVA infection and clinical symptoms, confirming that ANTXR1 acts as a receptor for the virus.

## Introduction

Senecavirus A (SVA), also known as Seneca Valley virus (SVV), is a non-enveloped, positive-sense, single-stranded RNA virus belonging to the genus Senecavirus in the family *Picornaviridae*^1^. The prototypic member of the group, Seneca Valley virus-001 (SVV-001), possesses a genome of 7200 nucleotides, flanked by 5ʹ and 3ʹ untranslated regions. The intervening sequence codes for a single 2181 amino acid polyprotein, which is proteolytically cleaved to yield four capsid-associated proteins and seven nonstructural proteins^2^. SVV-001 was first identified in 2002 as a contaminating virus in PER.C6 cells infected with an adenovirus 5 expression vector. In 2008, Hales et al. sequenced the unknown virus and named it SVV-001^1^. Within the human medical field, SVV is being used as an oncolytic agent that selectively targets tumor cells^3^.

SVA has been isolated from swine herds in the United States, China, Brazil, Canada, Colombia, and Thailand^4–6^. The first case of acute infection in North America occurred in 2007 when a vesicular disease outbreak was observed in a trailer of 187 Canadian market hogs^7^. Clinical signs included vesicular lesions on the snout and coronary bands. Because the lesions tested negative for foot and mouth disease virus (FMDV) and swine vesicular disease virus (SVDV), the syndrome was named idiopathic vesicular disease (IVD). The first confirmed case of SVA in the United States was reported in 2010 in Indiana^8^. Between 2014 and 2015, the number of SVA cases in the United States increased from 2 to over 230, demonstrating that SVA is a growing concern for producers^4,9^. Experimental infection studies confirmed the ability of SVA to cause vesicular lesions^4,10,11^; however, SVA infection can be present in pigs without overt signs of clinical disease. Since vesicular lesions caused by SVA are clinically indistinguishable from lesions caused by FMDV and SVDV, the appearance of IVD results in herd closure followed by a foreign animal disease investigation. Today, the prevalence of SVA on swine farms in the United States is estimated to be 43% in grow-finish pigs and 76% on sow farms^12^. In addition to causing IVD, SVA is associated with increased mortality in neonatal piglets^5,13,14^.

A loss-of-function screen, incorporating a lentivirus-expressed genome-wide CRISPR knockout (GeCKO) library, identified the *ANTXR1* gene as required for SVA infection of a haploid human cell line, HAP1, and other routes of SVA entry, if any, are unknown^15^. Knockout (KO) of the *ANTXR1* gene resulted in the resistance of permissive cell lines; and non-permissive cell lines, such as SCLC H69 and H146, are made permissive for SVA after transfection with a plasmid expressing *ANTXR1* cDNA. The porcine *ANTXR1* gene encodes a 564 aa protein, anthrax toxin receptor 1 (ANTXR1), also known as tumor endothelial marker 8 (TEM8). ANTXR1 binds the protective antigen protein of anthrax toxin; however, anthrax toxin receptor 2 (ANTXR2) has a greater binding affinity for the toxin^16^. The type I surface glycoprotein possesses a single transmembrane domain, and the increased abundance of ANTXR1 in tumors is responsible for permitting SVA replication and its oncolytic activity^17^. Besides binding anthrax toxin and SVA, ANTXR1 also binds type I and VI collagens, mediating interactions between cells and the extracellular matrix^18,19^. In humans, mutations in *ANTXR1* result in a rare disease known as growth retardation, alopecia, pseudoanodontia, and optic atrophy (GAPO) syndrome, as well as a reduced lifespan^20^. The purpose of this study was to evaluate the biological relevance of ANTXR1 in SVA pathogenesis in its natural host by infecting *ANTXR1* KO pigs with a pathogenic field strain of SVA.

## Methods

### Ethical use of virus and animals in research

The use of animals and viruses was in accordance with the Federation of Animal Science Societies (FASS) Guide for the Care and Use of Agricultural Animals in Research and Teaching, the USDA Animal Welfare Act, and Animal Welfare Regulations, Biosafety in Microbiological and Biomedical Laboratories, 5th edition. Pigs were humanely euthanized by intravenous pentobarbital injection according to American Veterinary Medical Association Guidelines on Euthanasia. Prior to initiating the study, all activities involving the use of virus and animals were approved by Institutional Animal Care and Use Committee (IACUC) and Institutional Biosafety Committee at Kansas State University (#1079), the IACUC at University of Missouri (#8813), and by the IACUC and IBC committees of Midwest Veterinary Services/Central States Research Centre (#AC21009P). The study was carried out in compliance with the ARRIVE guidelines.

### Generation of ANTXR1 KO pigs

Generation of the ANTXR1 KO pigs by using CRISPR/Cas9 is previously described by Whitworth et al.^21^. Single guide RNAs (sgRNAs) were designed in exon 1 of the *ANTXR1* gene (GenBank accession: NC_010445.4). Four 20 bp guides were designed adjacent to a Streptococcus pyogenes (Spy) protospacer adjacent motif (PAM) according to the Zhang Lab CRISPR Design^22^. Two pairs of guide sequences were located downstream from the start codon in exon 1 (Table S1).

### In vitro transcription of sgRNAs

Template guide DNA was synthesized by Integrated DNA Technologies (idtdna.com, Skokie, IL, USA) as a gBlock. For in vitro transcription, a T7 promoter sequence was added upstream of the guide. Each gBlock was diluted to a final concentration 0.1 ng/µL and amplified with gBlock Forward (5ʹ-ACTGGCACCTATGCGGGACGAC) and gBlock Reverse (5ʹ-AAAAGCACCGACTCGGTGCCAC) primers by using Q5 High-Fidelity DNA Polymerase (New England Biolabs, Ipswich, MA, USA) following standard procedures. PCR amplification included an initial denaturation of 98 °C for 1 min followed by 35 cycles of 98 °C (10 s), 68 °C (30 s), and 72 °C (30 s). Each PCR-amplified gBlock was purified by using a QIAGEN (Valencia, CA, USA) PCR purification kit following standard procedures. The purified gBlock amplicons were used as templates for in vitro transcription with the MEGAshortscript T7 Transcription Kit (Ambion, ThermoFisher, Grand Island, NY, USA). *Cas9* mRNA with the 5ʹ cap and polyadenylation signal was purchased from Sigma (St. Louis, MO, USA). Total single guide RNA (sgRNA) and *Cas9* mRNA were diluted in nuclease-free water and combined to final concentrations of 20 ng/µL and 20 ng/µL, respectively. Mix 1 contained sgRNA guides 58 and 99 with *Cas9* mRNA. Mix 2 contained sgRNA guides 64 and 101 with *Cas9* mRNA.

Preparation of embryos and injection of guide pairs were as described by Whitworth et al.^21^. Guide pairs Mix 1 or Mix 2 were co-injected as individual treatments into the cytoplasm of presumptive zygotes at 14 h post-fertilization. Injected zygotes were placed into MU2 for culture until day 5 or 6^23^. To assess blastocyst development and editing efficiency, three replicates of injections were performed for Mix 1 and Mix 2. Development to the blastocyst stage on day 6 for non-injected controls, Mix 1-injected, and Mix 2-injected groups were 33.3 ± 3.3%, 20.7 ± 3.4%, and 18.5 ± 5.4%, respectively. Embryo transfers were performed at the University of Missouri Swine Research Complex. Fifty blastocysts and morulae were loaded into a tomcat catheter and surgically transferred into the ampullary-isthmic junction of a cycling gilt on days 3 or 4 after standing estrus. Pregnancy was determined by heat checking and monitoring by ultrasound after day 25. Surrogates were checked weekly until farrowing. The sexes and birth weights of the piglets were recorded.

### Genotyping

Zona pellucidae of Day 6 blastocyst-stage embryos were removed, and genomic DNA was extracted as described by Whitworth et al.^21^. Briefly, genomic DNA was extracted from blastocyst-stage embryos by adding 6 μL of embryo lysis buffer (ELB) (40 mM Tris, pH 8.9, 0.9% Triton X-100, 0.9% Nonidet P-40, 40 μg/mL Proteinase K) and incubating at 60 °C for 45 min. Genomic DNA was isolated from Day 1 piglets by incubating a piece of ear skin in 300 μL of ELB at 56 °C for 4–5 h. Proteinase K was inactivated by incubating lysates at 85 °C for 10 min. Primers for the genotyping assay were *ANTXR1* Forward (5ʹ-GGTCCTGGCGACTTAGA) and *ANTXR1* Reverse (5ʹ-TGTATGCGGGACAACTTCT) which were located upstream of the start codon and within the first intron, respectively. The PCR amplicon was predicted to be 446 bp for the WT gene. PCR conditions consisted of 95 °C for 2 min, 38 cycles of 95 °C (30 s), 56 °C (40 s), 72 °C (30 s), and 72 °C for 1 min, and products were separated on a 2% agarose gel. PCR products were also subjected to Sanger sequencing with the *ANTXR1* Forward primer or cloned into pCR™4-TOPO vector (ThermoFisher) following standard procedures.

### Histology

One *ANTXR1* KO pig and one WT pig were humanely euthanized at one year of age for post-mortem examination at the University of Missouri Veterinary Medical Diagnostic Laboratory. Tissue samples were collected and fixed in 10% neutral buffered formalin. Fixed tissues were trimmed, processed by using a Sakura Tissue-Tek VIP 6 tissue processor (Torrance, CA, USA), embedded in paraffin, sectioned at 4 µm, and stained with hematoxylin and eosin for routine histopathology. In addition, Masson’s Trichrome stain and Alcian blue-PAS stain were used for the identification of connective tissue.

### Immunohistochemistry

For detection of ANTXR1 in lung tissue of WT and KO pigs, paraffin-embedded tissue sections were sectioned, deparaffinized, and enzymatically digested with Proteinase K. Sections were blocked with Dual Endogenous-Enzyme blocking reagent, avidin/biotin (Agilent, Santa Clara, CA, USA), in 1% blocking reagent (Roche, Basel, Switzerland) in TBS (100 mM Tris pH 7.5, 150 mM NaCl) with 1% Triton-X for 30 min at room temperature. Incubation was with rabbit anti-human TEM8 (clone 37, Abcam, Cambridge, United Kingdom) or IgG (Abcam) at 4 °C overnight, and detection was performed with Vectastain rabbit-IgG ABC HRP Kit (Vector Labs, Burlingame, CA, USA). Images were captured on Zeiss upright microscope and processed in Zen Blue software (Carl Zeiss, Thornwood, NY, USA).

### Cells and virus

Primary cultures of porcine fibroblast cells were prepared from tail snips of newborn pigs and placed at 4 °C in PBS with 10 μg/mL gentamicin. Fibroblast cells were isolated by incubating the tail snips at 37 °C in a humidified incubator with an atmosphere of 5% CO_2_ in air in DMEM supplemented with 15% fetal bovine serum (FBS), 10 μg/mL gentamicin, 400 U/mL collagenase (Sigma), and 4 Kunitz/μL DNase I (Sigma) for 3 h. Cells were grown until 90% confluent in a T75 flask, trypsinized, and frozen in FBS with 10% DMSO. To isolate individual colonies for confirming genotypes, cells were plated at clonal density (30 cells in a 100 mm dish) and grown for 7 days at 37 °C. Individual colonies were collected, genotyped, and frozen in liquid nitrogen.

The KS15-01 virus was originally isolated from a pig nasal swab sample submitted to the Kansas Veterinary Diagnostic Laboratory^10^. The virus was propagated on PK-15 cells with Minimal Essential Medium (MEM; ThermoFisher) supplemented with 2% horse serum (Sigma), antibiotics (100 units/mL of penicillin, 100 µg/mL of streptomycin, 0.25 µg/mL fungizone; ThermoFisher), and incubated at 37 °C and 5% CO_2_. For titration, the virus was serially diluted 1:10 in quadruplicate on confluent PK-15 cells in a 96-well tissue culture plate (BD Biosciences, San Jose, CA, USA). After two days, the wells were examined for the presence of cytopathic effect (CPE). The last well showing CPE was used as the titration endpoint, and the 50% tissue culture infectious dose (TCID_50_) per mL was calculated by using the method of Reed and Muench^24^. Modification of KS15-01 included the insertion of an EGFP gene fused with a Teschovirus 2A element between SVA genes 2A and 2B of the KS15-01 infectious clone and propagated as described by Chen et al.^10^. The EGFP-expressing virus, KS15-01-EGFP (GenBank accession No. KX349734), was used to infect fibroblast cells from WT and KO pigs. All images were captured at the same exposure. The growth kinetics in culture for the KS15-01 and the EGFP variant are the same^10^.

### Animal infection study

Pigs were infected with SVA isolate, KS15-01, according to the protocol described by Chen et al.^10^. Animal experiments were performed at the BSL-2 facility operated by Midwest Veterinary Services (Manhattan, Kansas, USA). WT and *ANTXR1* KO pigs (P generation) at 4 weeks of age or WT and *ANTXR1* IF pigs (F2 generation) at 8 weeks of age were maintained in the same room and allowed to co-mingle throughout the challenge period. After a one-week acclimation period, the pigs were infected with KS15-01, which was delivered intranasally as a 5 mL dose containing 10^8^ TCID_50_ of virus. Animals were examined daily for the presence of clinical signs. Serum, fecal swabs, and nasal swabs were collected at 0, 3, 7, and 10 days after infection. The experiment was terminated 10 days after infection, and the pigs were euthanized and necropsied.

### qRT-PCR of SVA nucleic acid

Viral RNA was extracted from serum, nasal swabs, or fecal swabs by using a MagMax 96-Viral RNA Isolation kit (Applied Biosystems, Foster City, CA, USA) according to the manufacturer’s instructions. The qRT-PCR was performed by using the EZ-SVA Real-Time RT-PCR detection kit from Tetracore (Rockville, MD, USA). Briefly, a 25 μL reaction was carried out by using 7 μL of extracted RNA, and all steps were performed according to the manufacturer’s instructions. Reverse transcription and amplification were performed on a CFX96 C1000 Thermal Cycler (Bio-Rad, Hercules, CA, USA), under the following conditions: reverse transcription at 48 °C for 15 min, initial denaturation at 95 °C for 2 min, and 45 cycles at 95 °C for 5 s and 60 °C for 40 s. A sample was considered negative a cycle threshold (Ct) ≥ 40.

### Total antibody and virus neutralization assays

Assays for the measurement of SVA-specific antibody and neutralizing activity in serum were performed according to Chen et al.^10^. For the measurement of SVA-specific antibodies, confluent PK-15 cells on a 96-well plate were infected with SVA KS15-01 and fixed with 80% acetone the next day. Beginning with an initial dilution of 1:5 in PBS with 5% goat serum (PBS-GS), serum was diluted 1:2 on a 96-well plate. All samples were assayed on the same plate. After incubation at 37 °C for 1 h, the wells were washed with PBS, and bound antibody was detected with FITC-conjugated goat anti-porcine IgG (Novus Biologicals, Centennial, CO, USA), diluted 1:400 in PBS-GS. Titration results were reported as the last dilution of serum showing fluorescence.

For measurement of SVA neutralizing activity, serum samples were first heated to 56 °C for 30 min to inactivate complement activity. Samples (100 μL) were mixed with 100 μL of medium containing 200 TCID_50_ of KS15-01. After 1 h incubation at 37 °C, the wells containing the virus-serum mixture were transferred to a 96-well plate of confluent PK-15 cells and incubated at 37 °C. After overnight incubation, the cells were fixed with 80% acetone for 10 min and then stained with anti-SVA VP2-specific monoclonal antibody, mAb30-158, followed by staining with Alexa Fluor® 488 AffiniPure goat anti-mouse IgG (Jackson ImmunoResearch Inc., West Grove, PA, USA) as a secondary antibody. Neutralizing titer results were reported as the highest serum dilution showing 90% or greater inhibition of virus growth.

### Statistical analysis

Ct values on Day 7 after infection for serum, feces, and nasal swabs of WT and IF pigs were analyzed by a two-tailed Student’s t-test in R (version 3.6.1). Type I error rate was controlled at a level of 0.05.

## Results

### Modification of ANTXR1 in porcine embryos

Two sets of single guide RNA (sgRNA) pairs were designed within exon 1 to induce out-of-frame mutations and formation of premature stop codons. These sgRNA pairs were labeled Mix 1 and Mix 2 with sequences described in Table S1. Genotypes were determined for 55 embryos injected with Mix 1 and 62 embryos injected with Mix 2. Based on gel shift assays and Sanger sequencing, injections resulted in 77.7 ± 5.8% and 77.8 ± 2.8% editing efficiencies for Mix 1 and Mix 2, respectively. Of the edited embryos injected with Mix 1, 74.5 ± 7.6% of embryos exhibited apparent monoallelic editing and 25.5 ± 7.6% had biallelic editing. Of the edited embryos injected with Mix 2, 66.1 ± 5.9% of embryos exhibited apparent monoallelic editing and 33.9 ± 5.9% had biallelic editing.

### Genotypic and phenotypic characteristics of ANTXR1-edited piglets

Three embryo transfers were performed by using embryos injected with Mix 1 sgRNAs. However, no pregnancies were maintained. Embryo transfers performed with embryos injected with Mix 2 sgRNAs resulted in two successful pregnancies. Litter 1 contained 4 piglets, 3 females and 1 male (Table S2). Litter 2 had 5 piglets, 2 females and 3 males (Table S3). Three piglets from Litter 2 were crushed by the dam during the first 4 days after birth. The two surviving males from Litter 2 were maintained to puberty to assess their fertility for breeding and propagating alleles.

**Figure 1:**
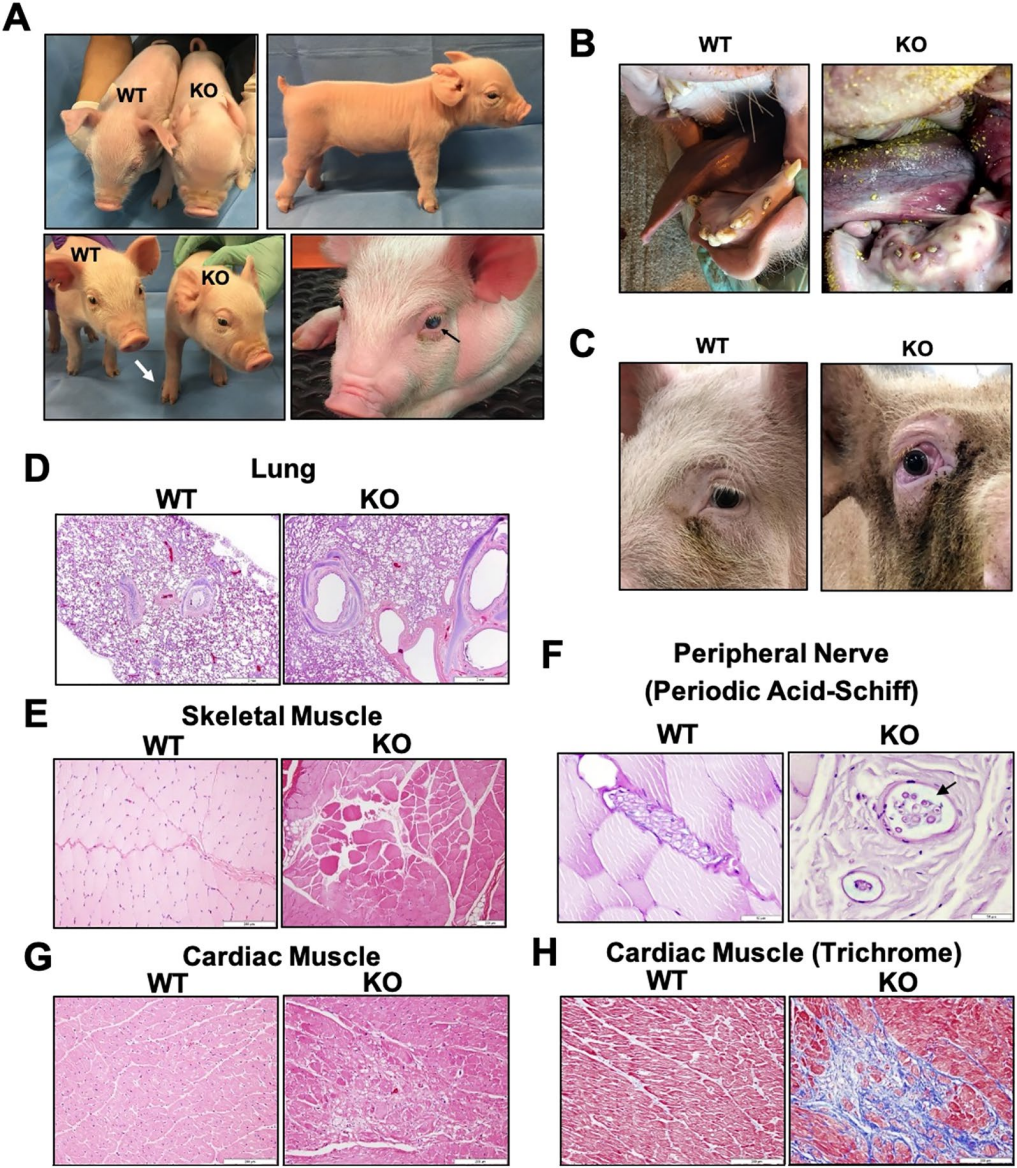
Anatomical and histological features of *ANTXR1* KO pigs. (**A**) The photographs show examples of anatomical abnormalities in *ANTXR1* KO pigs at two weeks of age. The upper left photograph shows an example of frontal bossing and shortened snout in the KO pig compared to a WT pig (left). The lower left photograph shows an example of a broadened stance (white arrow). The top right picture shows the post-legged rear legs of the KO pig. The bottom right picture shows an example of a KO pig with cataract (black arrow) in the left eye. (**B**) Presence of partial anodontia in the KO pig at one year of age. (**C**) Presence of excessive tissue development in the orbital socket with exophthalmos in the KO pig at one year of age. Representative images of hematoxylin and eosin (H&E) staining are shown for (**D**) lung, (**E**) skeletal muscle from the leg, and (**G**) cardiac muscle of WT and KO pigs at one year of age. (**F**) Periodic acid-Schiff staining was performed for skeletal muscle to show peripheral nerve degeneration with dilated fascicles filled with mucin (black arrow). (**H**) Masson’s trichrome staining was also performed for the cardiac muscle to show collagen accumulation (blue staining). Excessive collagen accumulation in the cardiac muscle was observed in several areas in the KO pig but not the WT pig. Scale bars are 2 mm for the lung, 200 μm for skeletal muscle and cardiac muscle, and 50 μm for the peripheral nerve.

The DNA sequences of five alleles obtained from Litter 1 are diagrammed in Figure S1 and Table S2. The KO alleles were the result of insertions and/or deletions in exon 1 which placed the resulting transcript out of frame and created premature stop codons. In addition to assessing their DNA sequence, pigs possessing a KO genotype were also confirmed based on expected physical appearance. Piglets with one or two wild type (WT) alleles showed no significant anatomical alterations and appeared normal at birth. Pigs possessing two KO alleles exhibited anatomical abnormalities, including frontal bossing, wide shoulders, short snouts, prominent facial skin folds, and post-legged rear legs (Fig. 1A). In addition, Piglet 1–4 was born with a cataract in the left eye (Fig. 1A; lower right). By one year of age, the KO pigs exhibited a more severe GAPO phenotype with partial anodontia and bilateral exophthalmos with shallow orbits (Fig. 1B,C).

Several tissues were obtained from a KO pig (2–1) from Litter 2 at one year of age as his fertility was too low for breeding (Fig. S1, Table S3). For comparison, tissues were obtained from a WT pig of similar age. Bronchi of the lungs from the KO pig showed multiplication of cartilage with two to four layers compared to a single layer in the WT pig (Fig. 1D). Skeletal muscle from the leg of the KO pig possessed degeneration and necrosis with hypereosinophilia, vacuolation, flocculation of muscle fibers, and interstitial edema (Fig. 1E). Nerves within the skeletal muscle of the KO pig exhibited degeneration with distended fascicles filled with mucin (Fig. 1F). Cardiac muscle also exhibited degeneration and necrosis in several areas (Fig. 1G). Trichrome staining for collagen accumulation showed the presence of fibrosis at several locations in cardiac muscle from the KO pig, while the WT pig did not demonstrate any fibrosis (Fig. 1H).

### SVA infection of ANTXR1 WT and KO fibroblast cells

**Figure 2:**
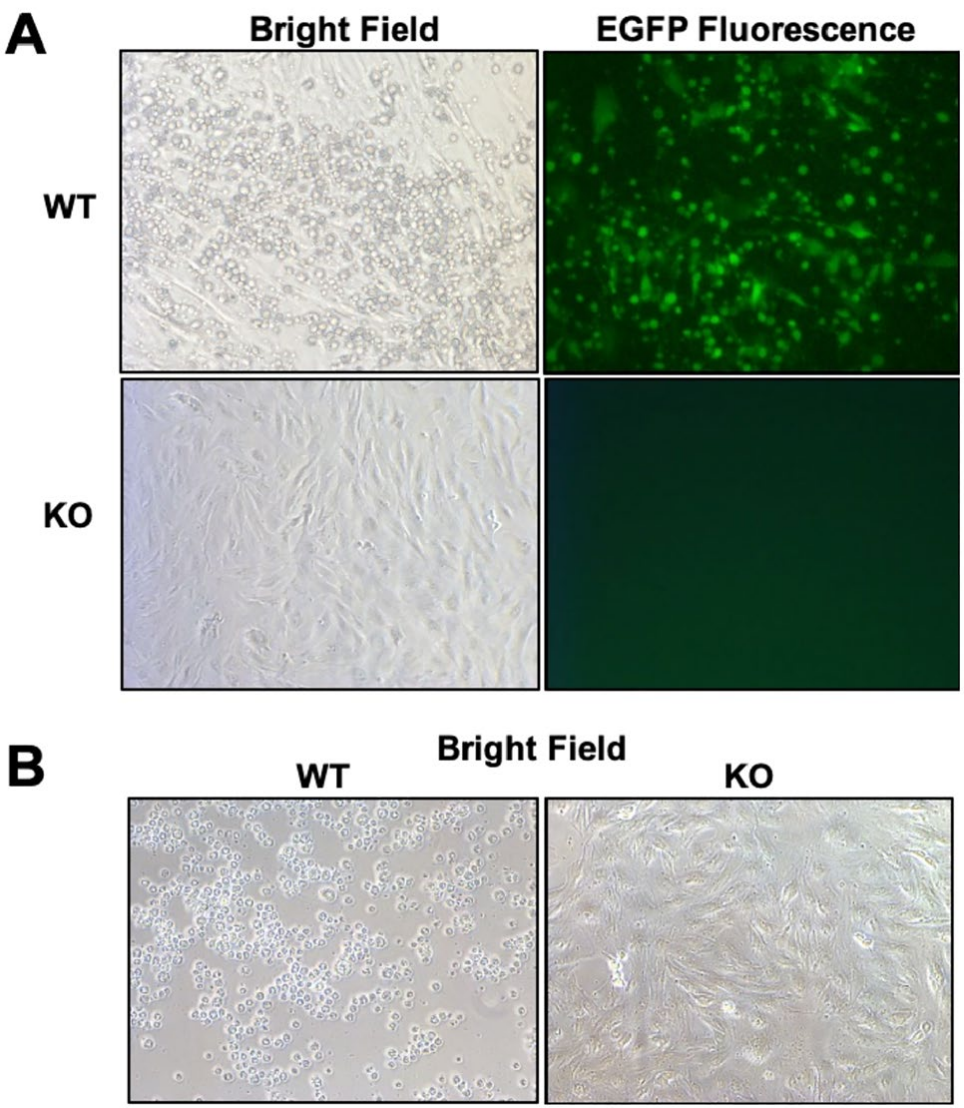
Infection of WT and KO cells with SVA-EGFP. Photomicrographs taken **(A)** 8 h and **(B)** 24 h after infection of fibroblast cells with SVA isolate, KS15-01-EGFP. The KO cells were generated from Pig 1–3 (52) (Fig. S1 and Table S2).

Primary fibroblast cells, derived from a WT and KO piglet, were infected in vitro with SVA isolate, KS15-01-EGFP. The KO cells were obtained from Piglet 1–3 (52), which possessed C and D KO alleles (Fig. S1, Table S2). Cells were infected at a multiplicity of infection (MOI) of 10 and examined 8 h later for the presence of cytopathic effect (CPE) and expression of EGFP fluorescence. Eight hours after infection reflects a time point when fluorescence is easily observable. By 24 h, fluorescence disappears because the EGFP leaks out of the dead cells. Cells in the WT culture exhibited CPE characteristic of SVA infection, including cell rounding and detachment from the monolayer (Fig. 2A,B). Fluorescence microscopy revealed that the rounded cells showed green fluorescence. In contrast, the cells from the KO pig showed no evidence of CPE or fluorescence. Consistent with previous studies, these results confirmed that *ANTXR1* KO cells are resistant to SVA infection^15^.

### Clinical outcomes, viremia, and immunological responses during SVA infection of ANTXR1 KO pigs

Two WT pigs from an age-matched litter, 54 and 59, and two KO pigs, 52 and 53 (Table S2), were infected with the KS15-01 isolate. Between six and eight days after infection, the WT pigs showed characteristic coronary band lesions on the feet (Fig. S2), which disappeared by nine days after infection. These observations were consistent with our previous study of pigs infected with the KS15-01 isolate^10^. The two KO pigs showed no evidence of vesicular lesions or other disease-associated clinical signs.

**Figure 3:**
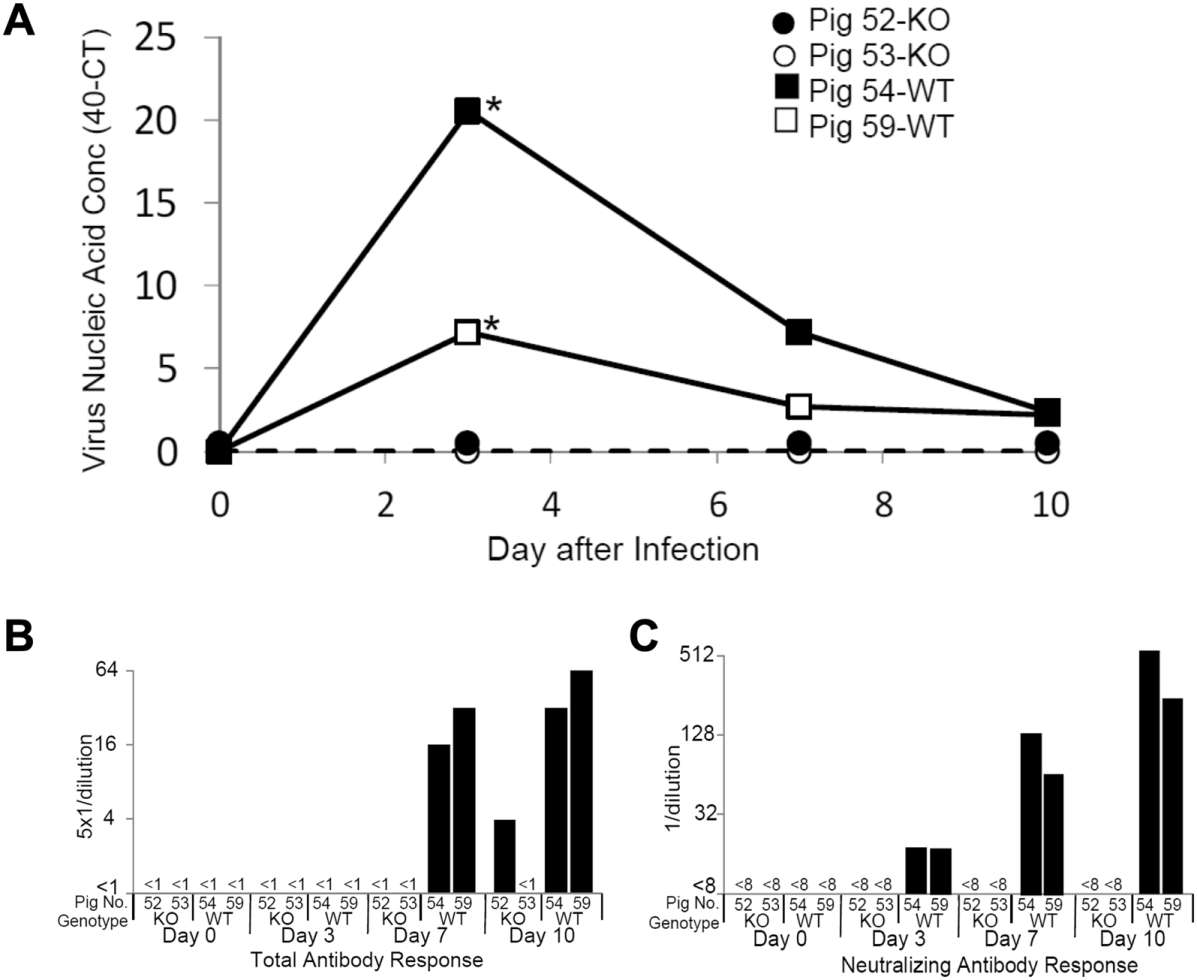
Circulating SVA nucleic acid and SVA-specific antibody responses in WT and KO pigs during the SVA challenge. (**A**) The concentration of nucleic acid is shown as the Ct value of the sample subtracted from 40, the Ct value cutoff. An asterisk identifies a serum sample that was positive for virus isolation on PK15 cells. (**B**) Total antibody response as measured by IFA. Serum, beginning with an initial dilution of 1:5, was serially diluted and incubated with SVA-infected PK-15 cells. (**C**) Virus neutralizing results for serum serially diluted 1:2. Dilutions less than 1:8 showed cytotoxicity on PK-15 cells. Results are shown for individual pigs.

Virus in sera was measured by using a commercial SVA qRT-PCR assay and by virus isolation of PK-15 cells. All pigs were negative for SVA by qRT-PCR prior to infection (Fig. 3A). The WT pigs produced a pattern of viremia typical for SVA infection by the KS15-01 isolate^10^. Ct values showed that both WT pigs were productively infected, with peak viremia appearing at three days after infection. Importantly, Day 3 samples from the WT pigs were positive for SVA by virus isolation (Fig. 3A). By the termination of the study, the Ct values for the WT pigs had returned to near background levels. All serum samples from the two KO pigs were negative for detectable amounts of viral nucleic acid (Ct > 40) and were negative by virus isolation. Even though the KO pigs were constantly exposed to virus shed by the two WT pigs, the KO pigs remained negative for detectable amounts of SVA in their sera (Fig. 3A).

Infection of the WT pigs was confirmed by the appearance of SVA-specific total antibody and by virus neutralization in the sera. The WT pigs seroconverted by Day 7 after infection and remained seropositive (Fig. 3B). KS15-01 neutralizing activity in the WT pigs was detected on Day 3 and continued to increase until the end of the study (Fig. 3C). Only one of the KO pigs showed evidence for SVA-specific antibodies, which appeared at the end of the study on Day 10. All samples from the KO pigs were negative for neutralizing activity (titer < 8).

### Post-challenge analyses of fecal swabs, nasal swabs, and tissues of ANTXR1 KO pigs

**Figure 4:**
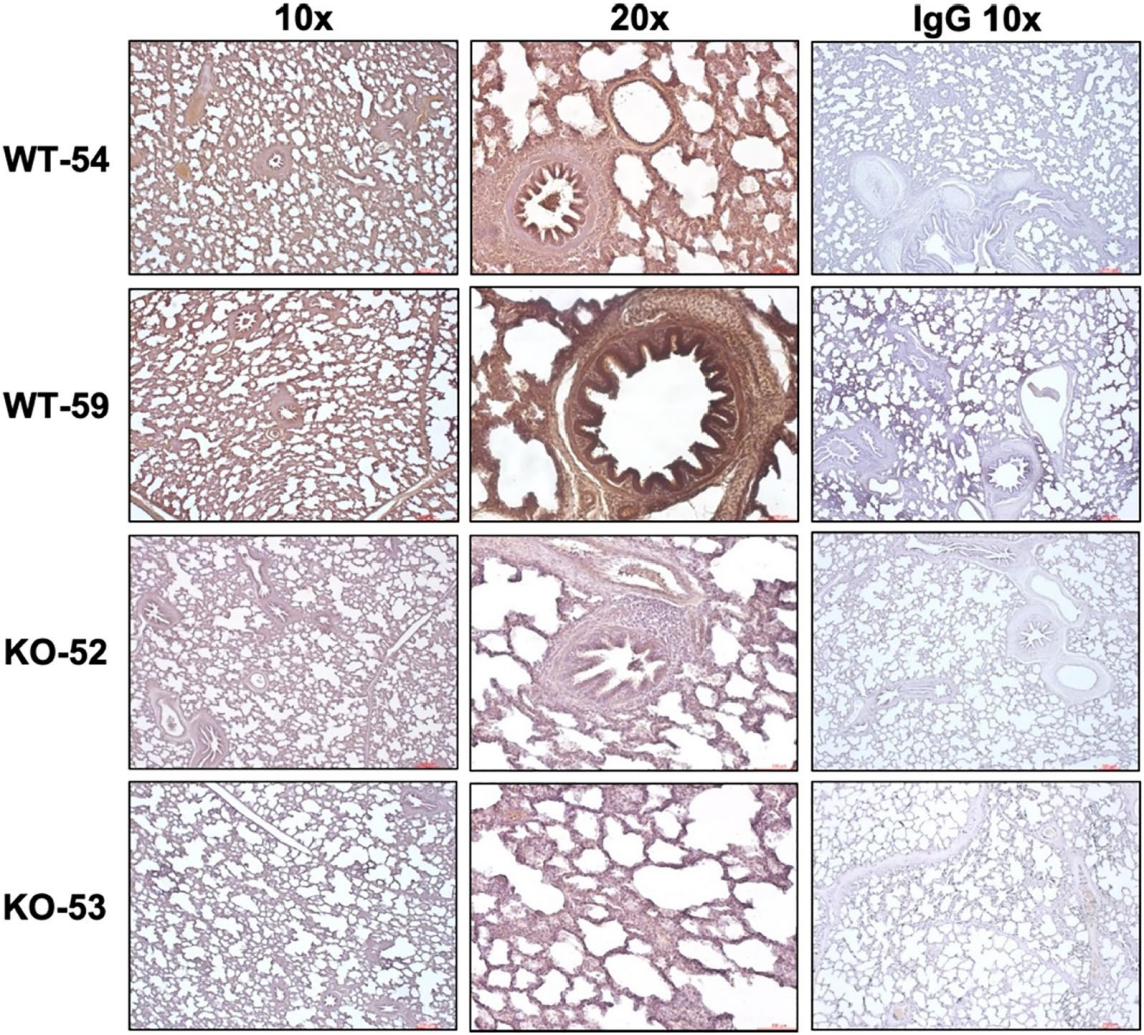
ANTXR1 detection in lung tissue from WT and KO pigs after the SVA challenge. Immunohistochemistry for ANTXR1 in lung sections for WT (54 and 59) and KO (52 and 53) at 10 × and 20 ×. Negative control sections incubated with rabbit IgG are shown to the right. Scale bars are 200 μm for 10 × and 100 μm for 20 ×.

Infection of pigs with KS15-01 results in relatively large quantities of virus shed in feces and nasal excretions^10^. Even though the KO pigs showed no evidence of virus in sera (Fig. 3A), viral nucleic acid was detected in feces, nasal swabs, and tissues. Overall, the Ct values for the WT pigs were lower compared to the results obtained from the KO pigs, indicating higher levels of viral shedding (Table 1). The greatest amount of viral nucleic acid for the KO pig samples was observed in the Day 3 fecal swabs (Ct > 31). For KO pig 52, viral nucleic acid was amplified from a nasal swab, as well as for lung and tonsil. Similar results were obtained for KO pig 53, except that viral nucleic acid was not detected in the lung. All samples from KO pigs were negative by virus isolation; whereas, fecal swabs taken from the WT pigs on Day 3 were positive by virus isolation (Table 1). Immunohistochemical analysis for detection of ANTXR1 protein in the lung tissue after the SVA challenge revealed high abundance in the WT pigs and little to no presence in the KO pigs (Fig. 4).

**Table 1:** A qRT-PCR results for fecal-nasal swabs and tissues from the first SVA challenge.

Pig	Day after infection						
	Feces				Nasal	Lung	Tonsil
	0	3	7	10	10	10	10
52-KO	> 40	31.3	34.2	37.1	38.3	33.3	38.3
53-KO	> 40	33.1	36.7	37.4	37.2	> 40	39.2
54-WT	> 40	27.2^†^	29.4	34.5	30.7	28.2	30.7
59-WT	> 40	27.1^†^	32.5	> 40	30.9	22.6	30.9

^†^Positive for SVA by virus isolation.

PCR results are presented as Ct values.

### Generation of piglets with a homozygous in-frame (IF) mutation in the N-terminus of ANTXR1

**Figure 5:**
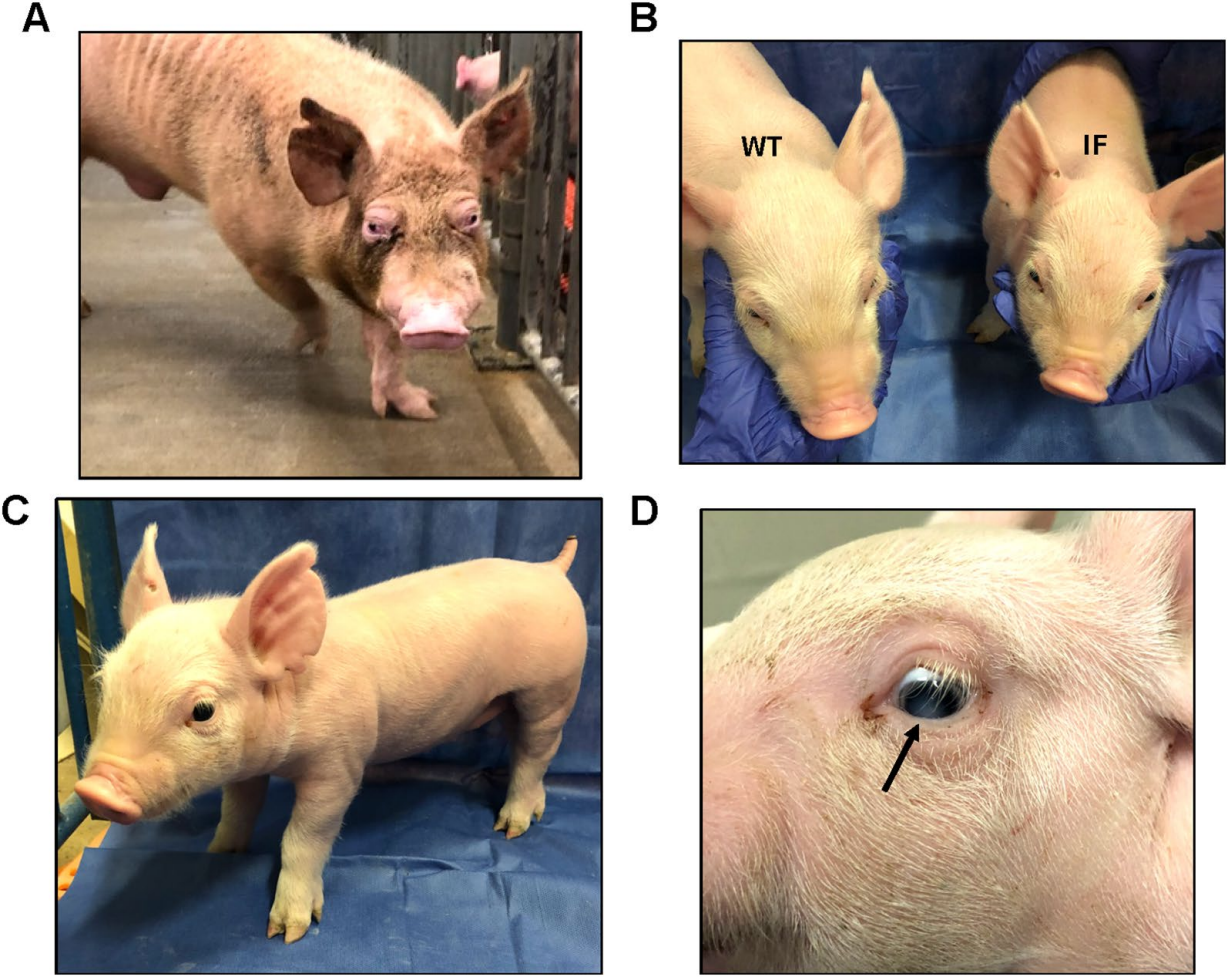
Gross anatomical features of *ANTXR1* in-frame (IF) pigs for the second SVA challenge. (**A**) The founder boar who was used for breeding was mosaic for edits in *ANTXR1* with two knockout alleles and one in-frame (IF) allele (Figure S3), and he exhibited a strong GAPO phenotype. After breeding to propagate his alleles, only the IF mutation was detected in progeny, and piglets were produced that were homozygous for this mutation. As shown, these piglets exhibited (**B**) frontal bossing and shorter snouts and (**C**) a post-legged stance as compared to WT piglets from the same litter. (**D**) Two homozygous IF piglets also had cataracts at birth (black arrow).

One founder boar (2–2) from Litter 2 was confirmed to be mosaic with three alleles detected after genotyping; two of which generated premature stop codons (Fig. S3A), and one was an in-frame (IF) mutation that was predicted to alter a region of 13 amino acids in the N-terminus of *ANTXR1* (Fig. S3A,B). The GAPO phenotype in the boar became very prominent with age (Fig. 5A), while all KO piglets had a more subtle phenotype (Fig. 1A). Furthermore, the boar had good fertility and was used to propagate the *ANTXR1* alleles. Surprisingly, only the IF mutation was detected in the progeny, indicating that this was the only allele that was incorporated into the germ cells; however, homozygous IF piglets, derived from intercrossing heterozygous IF parents, demonstrated the GAPO phenotype with frontal bossing, short snouts, post-legged stance, and cataracts (Fig. 5B–D). As such, it was not known if the homozygous IF piglets were resistant to SVA and were therefore subjected to in vitro and in vivo SVA challenges.

### SVA infection of ANTXR1 WT, KO, and IF fibroblast cells

**Figure 6:**
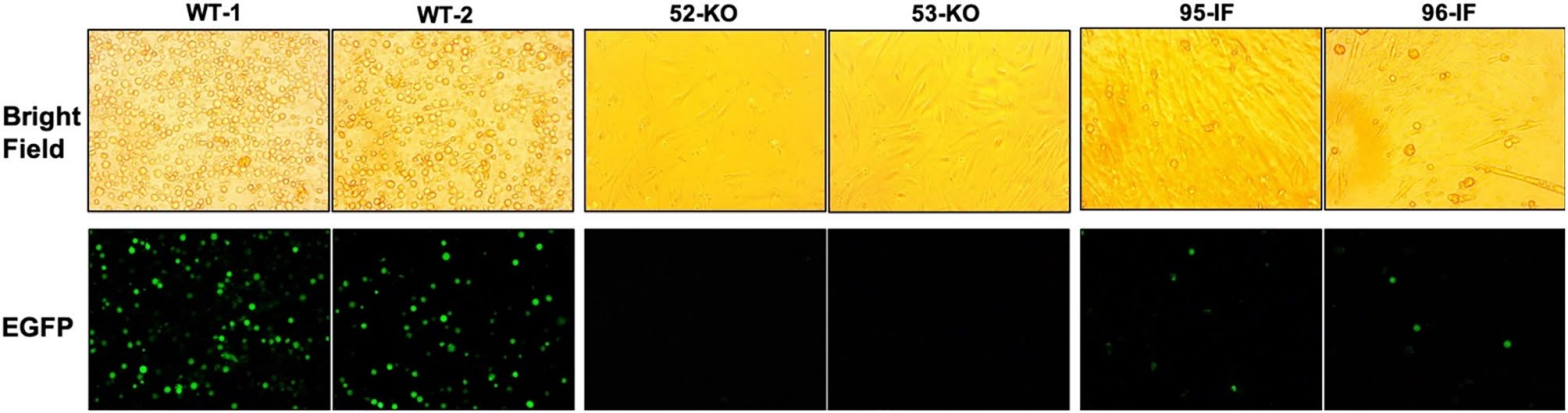
Infection of WT, KO, and IF cells with SVA-EGFP. Photomicrographs taken 8 h after infection of fibroblast cells with SVA isolate, KS15-01-EGFP, at the same exposure. KO cells were from the two pigs of the first SVA challenge, and IF cells were from two pigs of the second SVA challenge.

Primary fibroblast cells, derived from WT, KO, and IF piglets, were infected in vitro with KS15-01-EGFP as described above. The KO cells were obtained from the two piglets, 52 and 53, from the first challenge. The IF cells were obtained from piglets 95 and 96 from breeding the founder boar. Cells in the WT cultures exhibited CPE characteristic of SVA infection, including cell rounding and detachment from the monolayer (Fig. 6). Fluorescence microscopy revealed that the rounded cells showed green fluorescence. In contrast, cells from the KO piglets showed no evidence of CPE or fluorescence, while cells from the IF piglets showed a decrease in the number of fluorescent cells (Fig. 6).

### Detection of SVA nucleic acid in sera, fecal swabs, and nasal swabs of ANTXR1 IF pigs

Four WT and four IF piglets were infected with KS15-01. One of the IF piglets showed a vesicular lesion. However, none of the other pigs in this second study developed obvious vesicular lesions, a likely consequence of being older (approximately 8 weeks at the time of infection). WT piglets had detectable SVA nucleic acid in the sera, feces, and nasal swabs by Day 3 after infection (Table 2). The presence of SVA nucleic acid was detected in sera of IF piglets by Day 7; albeit, Ct values were higher in the IF compared to the WT piglets (*P* = 0.003). Moreover, SVA nucleic acid was detected in the fecal and nasal swabs of IF piglets on Day 7 (Table 2), pointing to a mitigated infection response.

**Table 2:** A qRT-PCR results for fecal-nasal swabs from the second SVA challenge.

Pig	Day after infection								
	Serum			Feces			Nasal		
	0	3	7^‡^	0	3	7	0	3	7
91-WT	> 40	34.0	28.4	> 40	> 40	25.3	> 40	36.6	21.9
93-WT	> 40	38.3	29.3	> 40	33.5	32.5	> 40	> 40	25.8
94-WT	> 40	34.1	26.4	> 40	35.4	24.4	> 40	> 40	28.9
98-WT	> 40	> 40	31.5	> 40	> 40	32.8	> 40	> 40	28.1
92-IF^†^	> 40	> 40	37.0	> 40	> 40	33.3	> 40	> 40	30.7
95-IF	> 40	> 40	33.3	> 40	> 40	31.6	> 40	> 40	29.9
96-IF	> 40	> 40	35.4	> 40	> 40	33.2	> 40	> 40	29.4
97-IF	> 40	> 40	34.6	> 40	> 40	34.1	> 40	> 40	26.0

PCR results are presented as Ct values.

^†^IF: Piglets homozygous for the in-frame mutation shown in Fig. 5.

^‡^Ct values for Day 7 Serum were statistically different between WT and IF pigs (*P* = 0.003).

## Discussion

ANTXR1 participates in a variety of seemingly unrelated activities, including the host response to infection with *Bacillus anthracis*, the regulation of collagen deposition in tissues, and as a receptor for SVA. Moreover, the upregulation of ANTXR1 in tumors makes SVA a convenient therapeutic treatment for the targeted killing of cancer cells. For this study, the biological relevance of porcine ANTXR1 as a receptor for SVA was evaluated by infecting pigs that possessed a deletion and/or insertion of nucleotides in the first exon of the *ANTXR1* gene. In addition to DNA sequencing, mutations in *ANTXR1* were confirmed by the appearance of anatomical and tissue abnormalities, similar to those found in KO mice and mutations in *ANTXR1* associated with the rare disease, known as GAPO syndrome, in humans^20,25^. In pigs, these changes included frontal bossing, short snouts, post-legged stance, partial anodontia, hair and skin abnormalities, and ophthalmic changes along with alterations in lung, muscle, and nervous system tissues, which become more prominent with age. Histological examination of skeletal muscle did not reveal differences between WT and *ANTXR1* KO mice^26^, but skeletal muscle degeneration and edema were observed in KO pigs at several locations. However, the age of the mice was not stated, which could influence the extent of degeneration. Importantly, cardiac muscle fibrosis and degeneration was present in numerous areas of the heart section from the KO pig and may contribute to the reduced lifespan of patients with GAPO syndrome^20^. One unique anatomical feature observed in this study was the appearance of cataracts in the eyes of KO and IF pigs, a phenotype that has so far only been reported in GAPO patients^27^. A mechanistic link between cataracts and ANTXR1 is not known. Thus, the *ANTXR1*-edited pigs provide a useful model for investigating GAPO syndrome in humans as the pigs more closely recapitulate the disease phenotype.

Senecavirus A continues to be an issue for swine producers as vectors, including mice and flies, are involved in the transmission of the virus within and between farms^4^, and SVA has been shown to be stable in several swine feed ingredients for up to a month^28^. Although vaccines are being developed, they have not conferred prolonged protection^29^; thus, warranting the need for new strategies, such as gene editing that have resulted in resistance to porcine reproductive and respiratory syndrome virus (PRRSV) and transmissible gastroenteritis virus (TGEV)^30,31^. In the present study, cells from an *ANTXR1* KO pig that were confirmed to have two KO alleles showed resistance to infection with SVA-EGFP. This supports the results of Miles et al. who demonstrated that cells possessing a KO of *ANTXR1* are resistant to infection with SVV-001^15^. Similar results were obtained for the *ANTXR1* KO pigs after infection with SVA. The design of the animal infection experiments included housing KO and WT pigs in the same pen, which allowed for constant exposure of the KO pigs to virus shed by the infected WT pen mates. Even though the KO pigs showed no evidence of circulating virus or disease, SVA qRT-PCR-positive results were obtained for feces, nasal swabs, tonsil, and lung samples from the KO pigs, and KO Pig 52 seroconverted on Day 10. Detection of SVA nucleic acid in fecal and nasal swabs of the KO pigs may have been due to contaminating virus shed from the WT pigs. Moreover, because the KO pigs were generated by injection of the CRISPR/Cas9 system into the zygote, unequal editing of the blastomeres may have occurred. This could have resulted in mosaicism that was not detected after genotyping and the potential for the presence of a low number of WT alleles that permitted reduced infection in KO Pig 52. Importantly, previous studies have shown that only one KO pig is required to demonstrate resistance to a virus^30,31^, and KO Pig 53 did not have clinical symptoms, presence of SVA nucleic acid in the serum or lung, nor produce any SVA-specific antibodies during the challenge. Thus, the negative responses by KO pig 53 to SVA in vivo and the resistance of KO fibroblasts from Pig 52 with a confirmed genotype to SVA infection in vitro were instrumental in verifying that ANTXR1 is the receptor for SVA in pigs.

Interestingly, we produced piglets that were homozygous for an in-frame (IF) mutation in the N-terminus that was predicted to alter a region of 13 amino acids immediately proximal to the signal peptide. These piglets also exhibited the GAPO phenotype, indicating that these amino acid changes may be sufficient to disrupt interactions with extracellular matrix molecules, or that the levels of ANTXR1 on the cell surface were reduced due to aberrant cleavage of the signal peptide. When cells from the IF piglets were challenged with SVA-EGFP, only a few became round and exhibited fluorescence, pointing to a decreased level of infection. After nasal inoculation with SVA, the IF piglets did have detectable but lower levels of circulating SVA nucleic acid by the end of the challenge, consistent with the in vitro results. While the regions of ANTXR1 responsible for binding collagen have not yet been mapped, studies have shown that 16 amino acids of ANTXR1 directly interact with SVA^32^. Therefore, it is possible that mutation of some of these key amino acids could block SVA without disrupting collagen binding. Furthermore, N-linked glycosylation of specific ANTXR1 residues has recently been shown to be critical for SVA entry into human cells^33^, thus revealing new targets for potentially blocking SVA infection without interfering with the normal physiological function of ANTXR1.

Overall, genetic disruption of *ANTXR1* confers protection against SVA infection and clinical symptoms in pigs. Moreover, we have identified a new mutation in the N-terminus of *ANTXR1* that leads to both a GAPO-like phenotype and decreased permissiveness of SVA in pigs. Further studies with more animals will be useful for discerning which residues or glycosylation sites on ANTXR1 are important for SVA infection; however, the *ANTXR1*-edited pigs will need to be reanimated from frozen cells by somatic cell nuclear transfer or semen by artificial insemination. Nevertheless, the information generated in this study has increased our understanding of virus-receptor interactions in pigs, which has applications for controlling SVA infections in swine herds across the world and for potentially modeling the oncolytic properties of SVA as a cancer therapeutic.

## Supplementary Information

Supplementary Information.

## Data Availability

The data that support the findings of this study are available upon request from the corresponding author.
