# Supplementary material for: Disruption of anthrax toxin receptor 1 in pigs leads to a rare disease phenotype and protection from senecavirus A infection
Source: Sci Rep. 2022 Mar 23;12:5009. doi: 10.1038/s41598-022-09123-x (PMC8943192; doi:10.1038/s41598-022-09123-x)
Supplement: Supplementary file 1 — Supplementary Information. [file 41598_2022_9123_MOESM1_ESM.pdf]

**Supplementary Information**

**Table S1. Single guide RNAs designed to target *ANTXR1*.**

| **Name** | **Sequence (PAM sequence in bold)** |
|---|---|
| Mix 1 Guide-58[†] | 58-CTTTTGTGCTCATCTGCGCC**GGG** |
| Mix 2 Guide-64 | 64-TGCTCATCTGCGCCGGGCAA**GGG** |
| Mix 1 Guide-99 | 99-GAAGGGGGTCCAGCCTGCTA**CGG** |
| Mix 2 Guide-101 | 101-AGGGGGTCCAGCCTGCTACG**GGG** |

[†] Number refers to position after start codon.

**Table S2. Genotypes of *ANTXR1*-edited piglets from Litter 1.**

| Litter-Pig† | Sex | Edit | Phenotype‡ | Allele 1§ | Allele 2 |
|---|---|---|---|---|---|
| 1-1 | F | Monoallelic | WT | A | B |
| 1-2 | F | No Edit | WT | A | A |
| 1-3 (52) | M | Biallelic | KO | C | D |
| 1-4 (53) | F | Biallelic | KO | C | E |

† Numbers in parentheses identify the pigs used in the SVA infection studies.

‡ Phenotype determined by the presence of anatomical features characteristic of *ANTXR1* KO pigs shown in Figure 1.

§ Diagram of each allele is shown in Figure S1.

**Table S3. Genotypes of *ANTXR1*-edited piglets from Litter 2.**

| Litter-Pig | Sex | Edit | Phenotype[†] | Allele 1[‡] | Allele 2 | Allele 3 |
|---|---|---|---|---|---|---|
| 2-1 | M | Biallelic | KO | F | G | ND[§] |
| 2-2 | M | Mosaic | KO | H | I | J |
| 2-3[¶] | F | Biallelic | KO | F | K | ND |
| 2-4[¶] | M | Mosaic | KO | A | C | F |
| 2-5[¶] | F | No Edit | WT | A | A | ND |

[†] Phenotype determined by the presence of anatomical features characteristic of *ANTXR1* KO pigs shown in Figure 1.

[‡] Diagram of each allele is shown in Figure S1.

[§] ND, not detected.

[¶] Died shortly after farrowing.

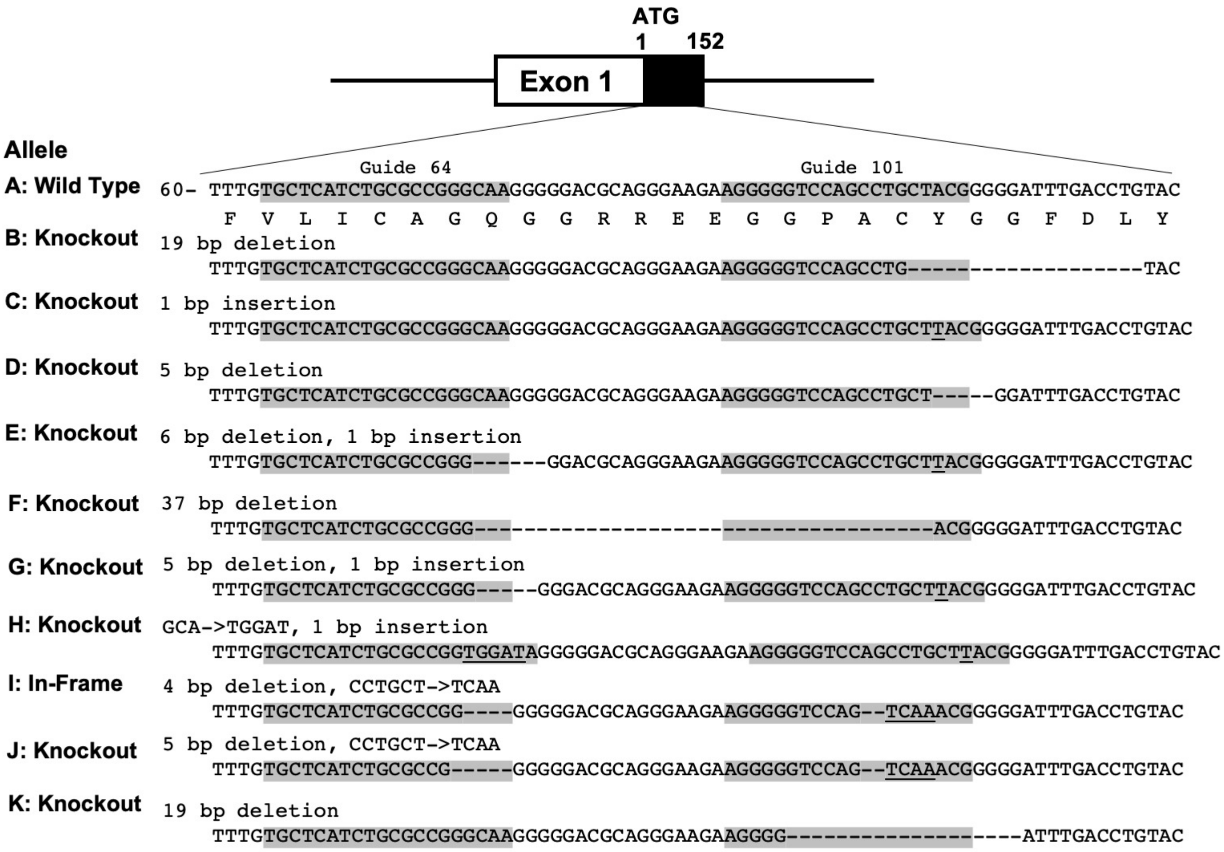


**Figure S1. Edits in exon 1 of *ANTXR1*.** Guides 64 and 101 shown in Table S1 were used to create piglets with edits in exon 1 of *ANTXR1*. The top diagram shows the *ANTXR1* non-coding region in exon 1 as white and the coding region in black, beginning with the ATG start codon. In the bottom diagram are the sequences of the different alleles generated by guides 64 and 101. Guide sequences are shaded. Encoded amino acids are shown below the Wild Type allele. A premature stop codon was generated by alleles with the Knockout description. A region of 13 amino acids was disrupted in the allele with the In-Frame description, but a stop codon was not generated. Dashed lines represent deletions, and underlined letters represent insertions.

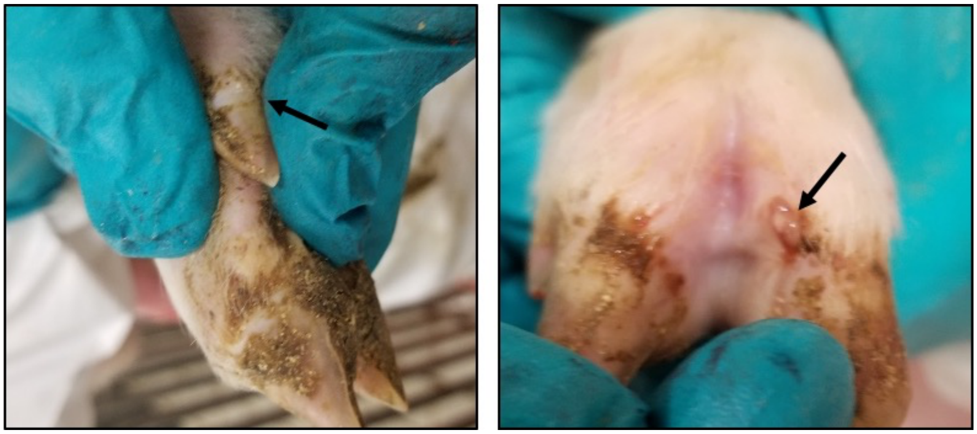

**Figure S2. Examples of hoof lesions in SVA-infected pigs.** Left and right pictures show the presence of vesicular lesions on the coronary bands of the WT pigs (54 and 59), identified by black arrows, at 6 to 8 days after infection. No lesions were observed on the *ANTXR1* KO pigs (52 and 53).

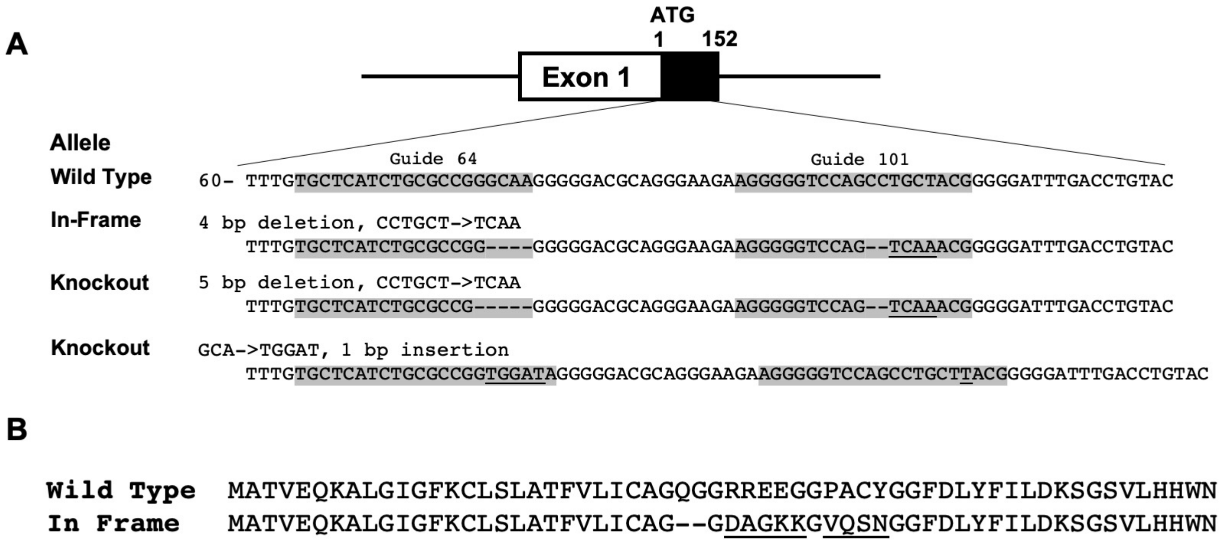


**Figure S3.** ***ANTXR1*** **exon 1 edits for the second SVA challenge.** One founder boar (2-2 from Litter 2) was maintained as a breeder with good fertility. **(A)** Three alleles (below Wild Type allele) were detected after genotyping an ear snip with one in-frame mutation and two knockout mutations that generated premature stop codons. Guide sequences (Table S1) are shaded. Dashed lines represent deletions, underlined letters represent insertions or nucleotide changes. **(B)** After breeding, only the in-frame mutation was detected in the progeny. The first 60 amino acids of the ANTXR1 protein are shown for wild type and the in-frame mutated alleles. Dashed lines represent deleted amino acids, and underlined amino acids signify changes compared to the wild type sequence.
